# Data on the organic matter characteristics of New Zealand soils under different land uses

**DOI:** 10.1016/j.dib.2018.10.016

**Published:** 2018-10-09

**Authors:** Qinhua Shen, Manuel Suarez-Abelenda, Marta Camps-Arbestain, Roberto Calvelo Pereira, Samuel R. McNally, Frank Kelliher

**Affiliations:** a School of Agriculture and Environment, Massey University, Private Bag 11222, Palmerston North 4442, New Zealand; b Departamento de Edafoloxía e Química Agrícola, Facultade de Bioloxía, Universidade de Santiago de Compostela, 15782 Santiago de Compostela, Spain; c Sustainable Production Portfolio, New Zealand Institute for Plant and Food Research Limited, Private Bag 4704, Christchurch, 8140, New Zealand; d AgResearch, Lincoln Research Centre, Private Bag 4749, Christchurch 8140, New Zealand

## Abstract

This article contains data related to the research article entitled “An Investigation of Organic Matter Quality and Quantity in Acid Soils as Influenced by Soil Type and Land Use” (Shen et al., 2018) [1]. The data was collected using a chemical fractionation scheme of soil organic matter (OM). This involved the separation of organic carbon (OC) fractions based on their solubility in (i) cold and hot water, (ii) 0.1 M sodium pyrophosphate (pH ~ 10), and (iii) 2% HF solution, and the residue remaining after the HF extraction. The OM in this residue, after treatment with 2% HF solution, was characterised using pyrolysis (Py)-GC/MS. This technique involves thermal decomposition of OM into various pyrolysis products, which are then chromatographically separated and determined by mass spectroscopy. This technique has been used to semi-quantify individual soil OM constituents so that in-depth information on soil OM molecular fingerprints is provided. This article presents a detailed dataset of physical-chemical characterization, OC fractions and OM molecular fingerprints of 62 soil samples for a range of soil orders (i.e., Allophanic, Brown, Gley, Pallic and Recent) and land uses (i.e., permanently grazed pasture, ungrazed/unmanaged grasslands, annual cropping) across New Zealand. Principal component analysis was used to investigate the relationships of different soil properties with OC fractions and OM chemistry so that the underlying mechanisms responsible for the differences encountered in OM quantity and quality between soil orders and land uses are understood.

**Specifications table**

**Table:** 

Subject area	*Earth Science*
More specific subject area	*Soil Chemistry*
Type of data	*Table and figure*
How data was acquired	*Soil physico-chemical characteristics were determined using conventional wet chemical analyses.*
	*Organic carbon fractions were extracted with cold + hot water-soluble (C*_*H2O*_*), sodium pyrophosphate (C*_*p*_*), and 2% HF (C*_*HF-residuum*_*and C*_*HF-mobile*_*).*
	*Soil organic matter pyrolysed with pyrolysis-GC/MS*
Data format	*Raw and processed*
Experimental factors	*Investigating the influence of soil properties (e.g., pH, Olsen P, clay content, short–range ordered materials and organo-Al complexes), soil orders (i.e., Allophanic, Brown, Gley, Pallic and Recent) and land uses (i.e., permanently grazed pasture, ungrazed/unmanaged grasslands, annual cropping) on soil OM quantity and quality.*
Experimental features	*A representative set of 45 soils (0–15 cm depth) were collected from New Zealand׳s major agricultural regions (Auckland, Waikato, Taranaki, Hawke׳s Bay, Canterbury, Southland). An additional subset of 17 Allophanic soils was used for specific analyses.*
Data source location	*New Zealand*
Data accessibility	*Data are available with this article*
Related research article	*Shen Q., Suarez-Abelenda M., Camps-Arbestain M., Calvelo Pereira R., McNally SR., Kelliher FM (2018). An investigation of organic matter quality and quantity in acid soils as influenced by soil type and land use. Geoderma. 328, 44–55**[1]*.

**Value of the data**

• The present data will help contribute to understanding the processes by which OM is protected in acid soils.
• The present data includes a comprehensive dataset of soil properties key to the preservation of OM in soils.
• The present data includes a comprehensive dataset of OC fractions, as obtained through chemical fractionation.
• The present data offers detailed information on molecular fingerprints of soil OM as obtained through Py-GC/MS.

## 1 Data

**Fig. 1:**
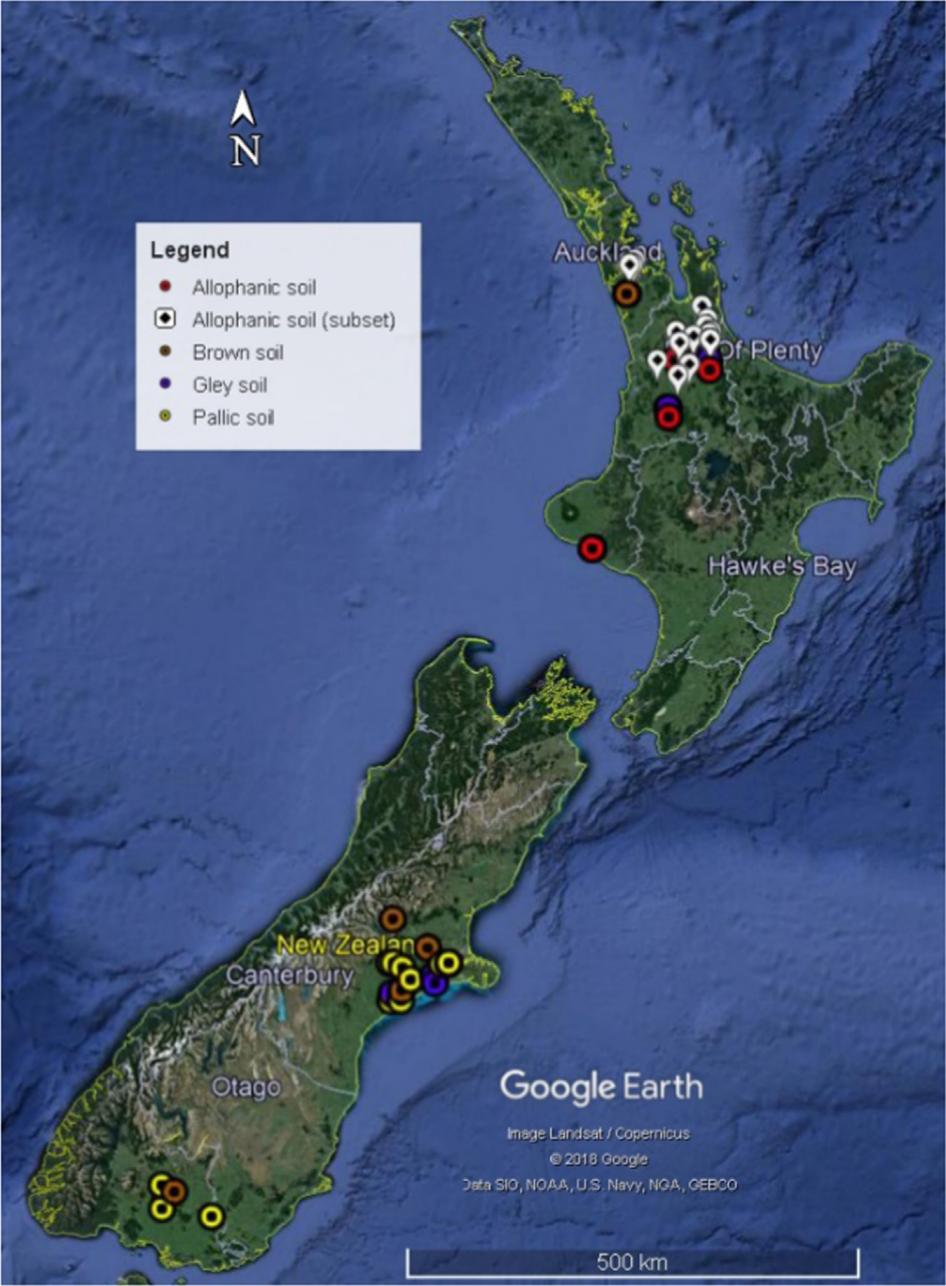
Map of soil sampling sites. This map was personalised from Google Earth 7.1.8.3036 (32-bit) using the built-in features (April 2018).

**Fig. 2:**
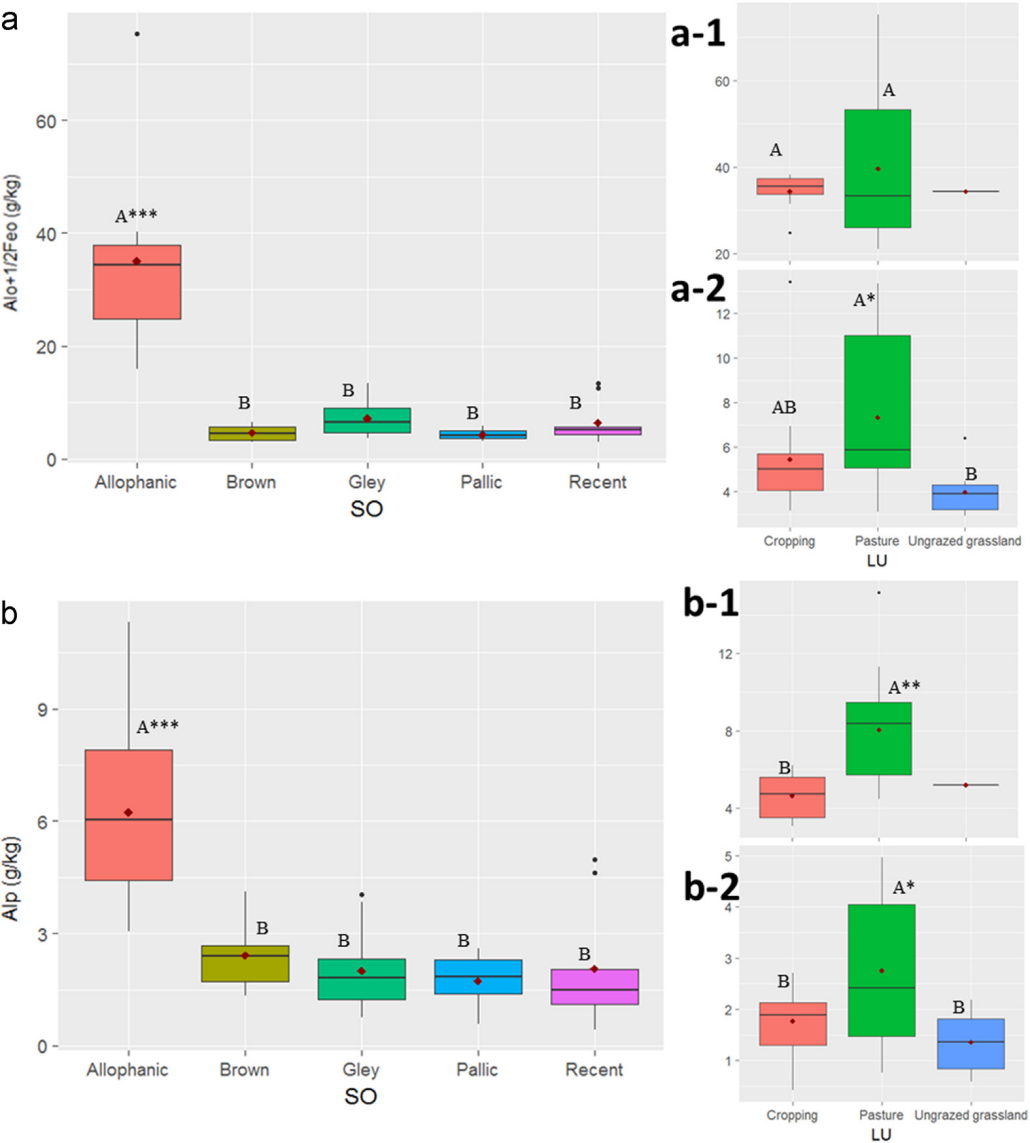
Abundance of short-range order constituents and organo-Al/Fe complexes (indicated by Al_o_ + ½Fe_o_ content) and Al cations associated with organic ligands (indicated by Al_p_ content) in the soils studied. Box plots showing: (a) Al_o_ + ½Fe_o_ content of all investigated soils categorised by soil order, and grouped to (a-1) Allophanic soils and (a-2) non-Allophanic soils under different land use; (b) Al_p_ content of all investigated soils categorised by soil order, and grouped to (b-1) Allophanic soils and (b-2) non-Allophanic soils under different land use. Each box-plot shows the following six elements: (1) mean (solid diamond in the box-plot); (2) median (solid bar in the box-plot); (3) 25th to 75th percentile (rectangular box); (4) minimum and maximum values (whiskers); (5) outlier values (solid black circles); and (6) significant differences (uppercase letters, one-way ANOVA followed by a TukeyHSD test, significant codes: ‘***’ 0.001, ‘**’ 0.01, ‘*’ 0.05, ‘.’ 0.1).

**Fig. 3:**
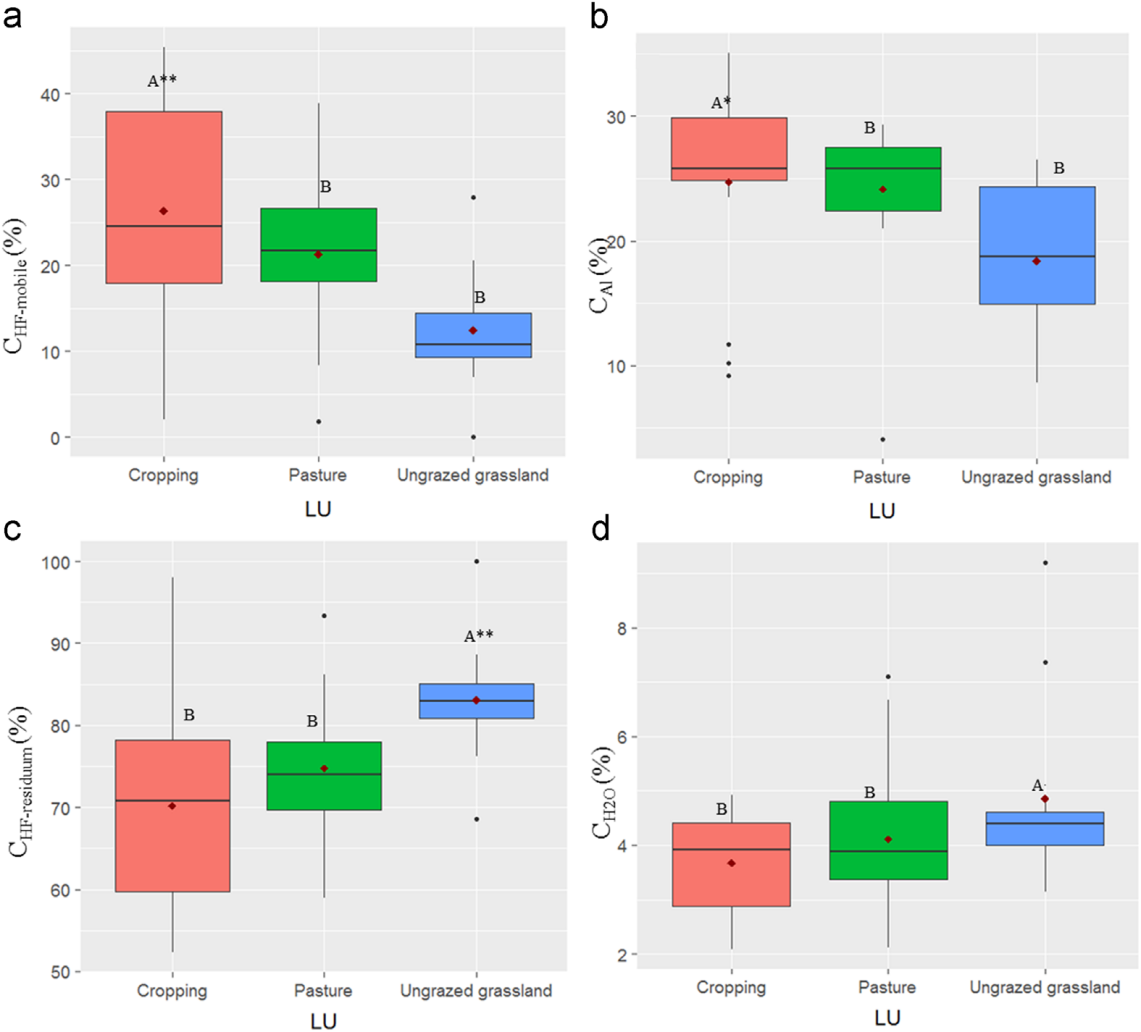
Relative values (%) of different C fractions of all soils studied (*n* = 45). Box plots showing the percentage of: (a) C_HF-mobile_, (b) C_Al_, (c) C_HF-residuum_, and (d) C_H2O_ grouped by land use. For box plots and abbreviations see Fig. 2. The sum of C_HF-mobile,_ C_HF-residuum_ and cold C_H2O_ is 100%.

**Fig. 4:**
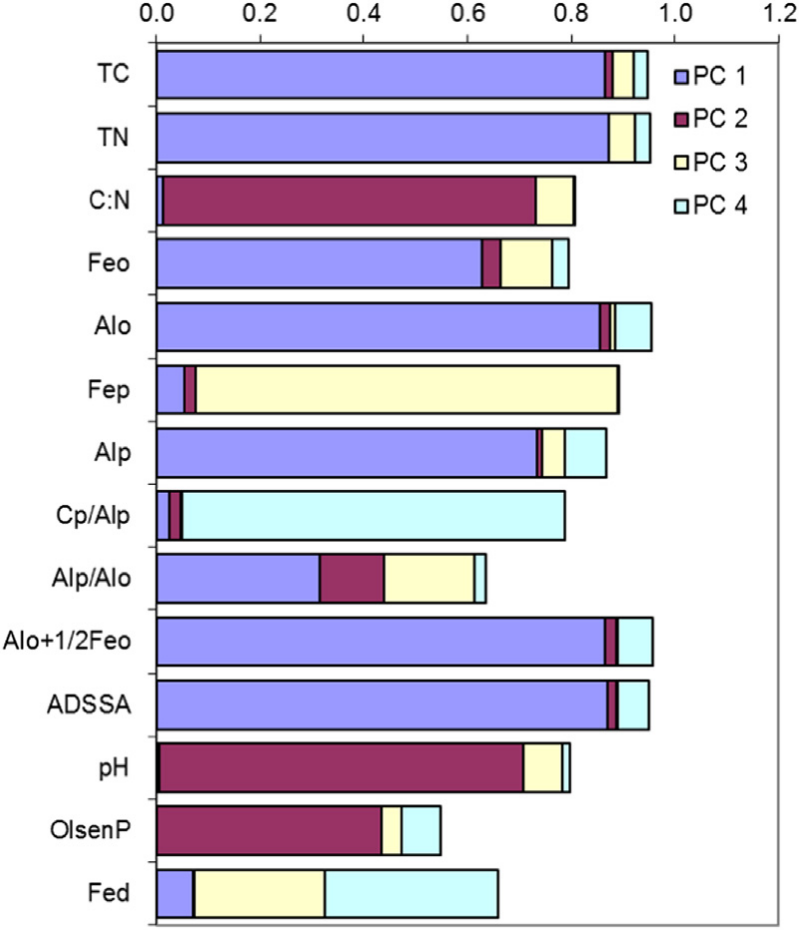
The communalities (square of loadings) of the PCA for the main soil properties considered, excluding C fractions.

**Fig. 5:**
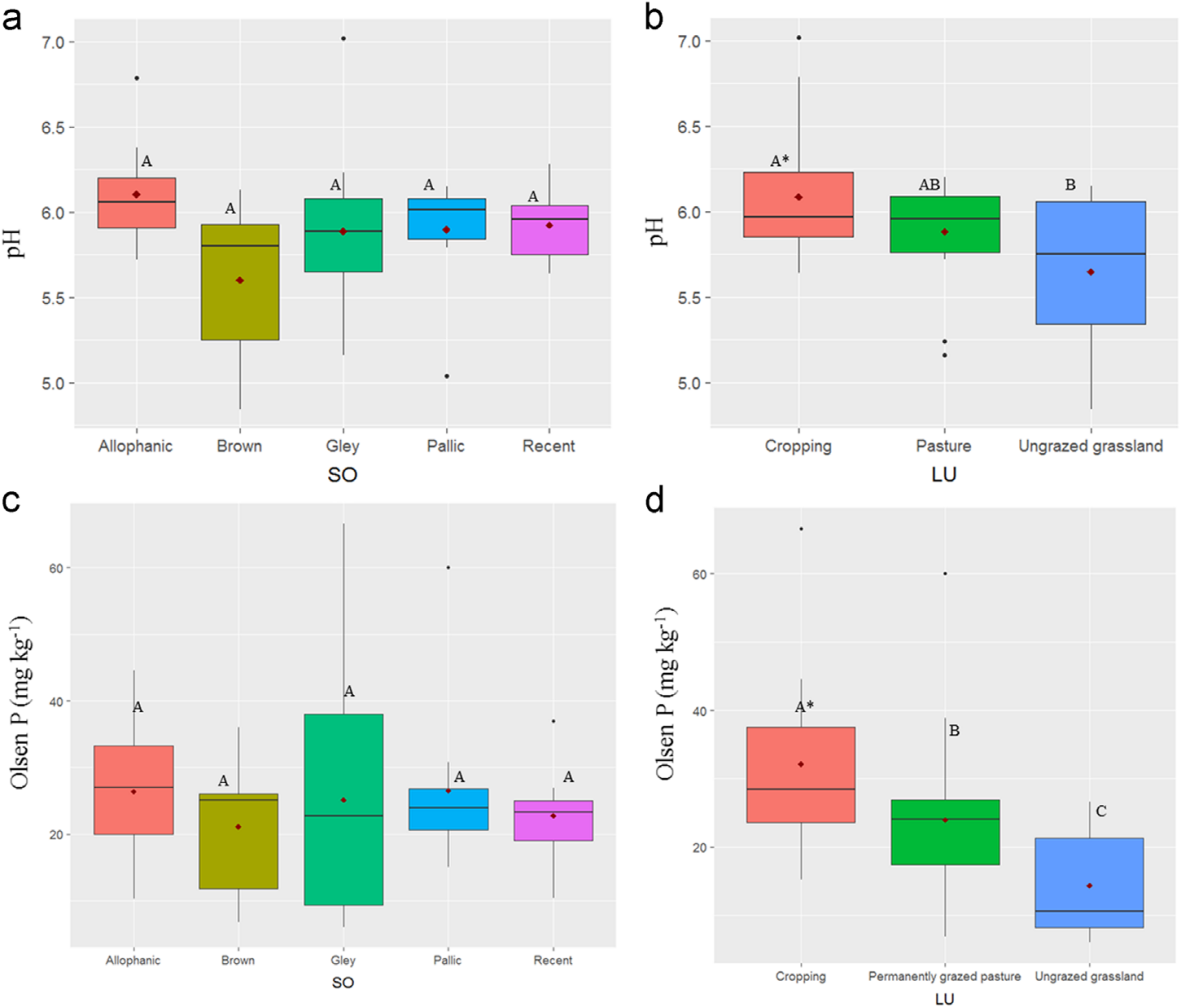
Values of pH and Olsen P of soil investigated. Box plots showing pH values of soils categorised by: (a) soil orders, and (b) land use, and Olsen P values of soils categorised by: (c) soil orders, and (d) land use. For box plots and abbreviations see Fig. 2.

**Fig. 6:**
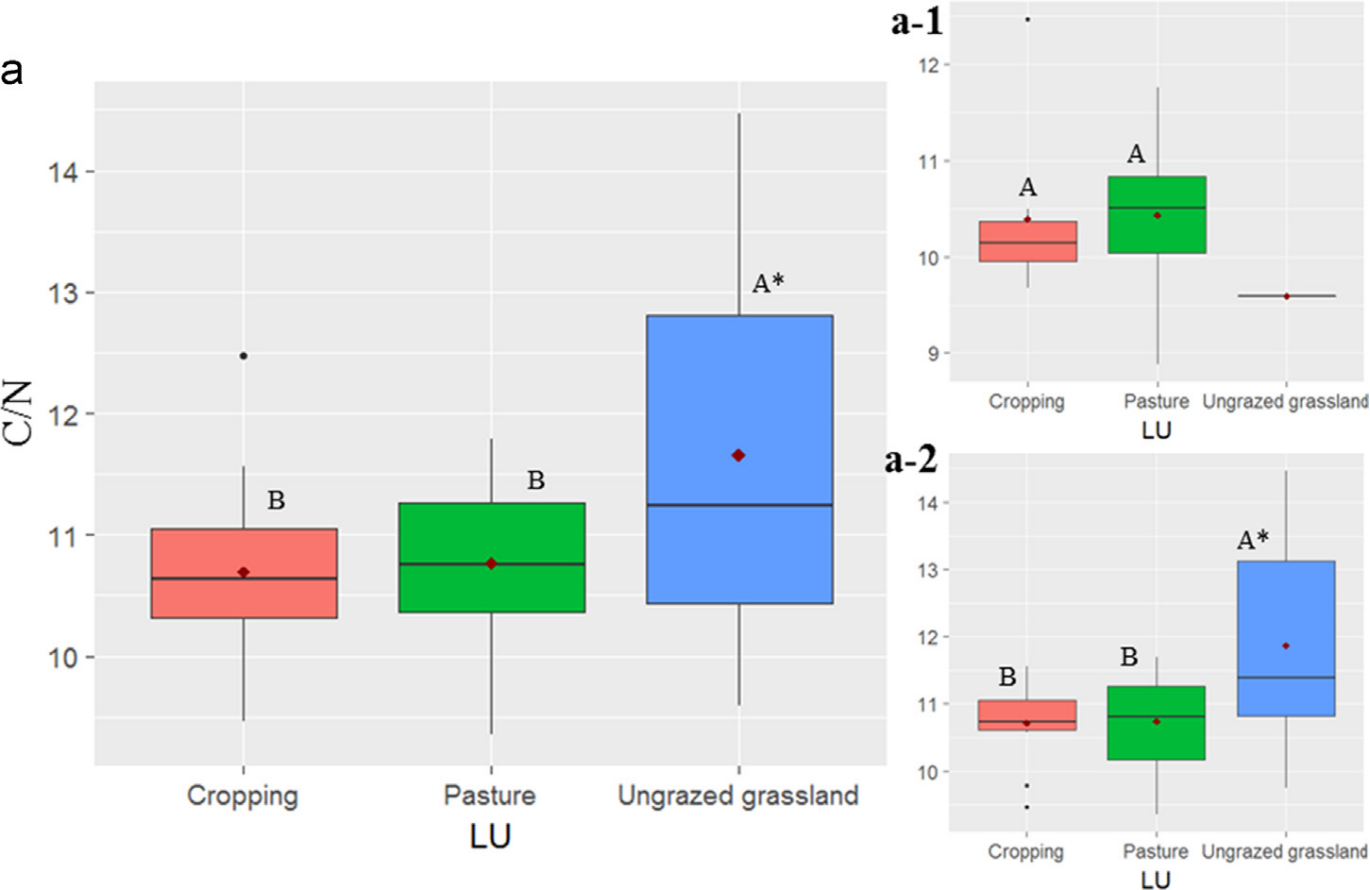
Ratio of C/N for the soils investigated. Box plots showing C/N ratio of: (a) all soils, (b) Allophanic soils, and (c) non-Allophanic soils under different land use. For box plots and abbreviations see Fig. 2.

**Fig. 7:**
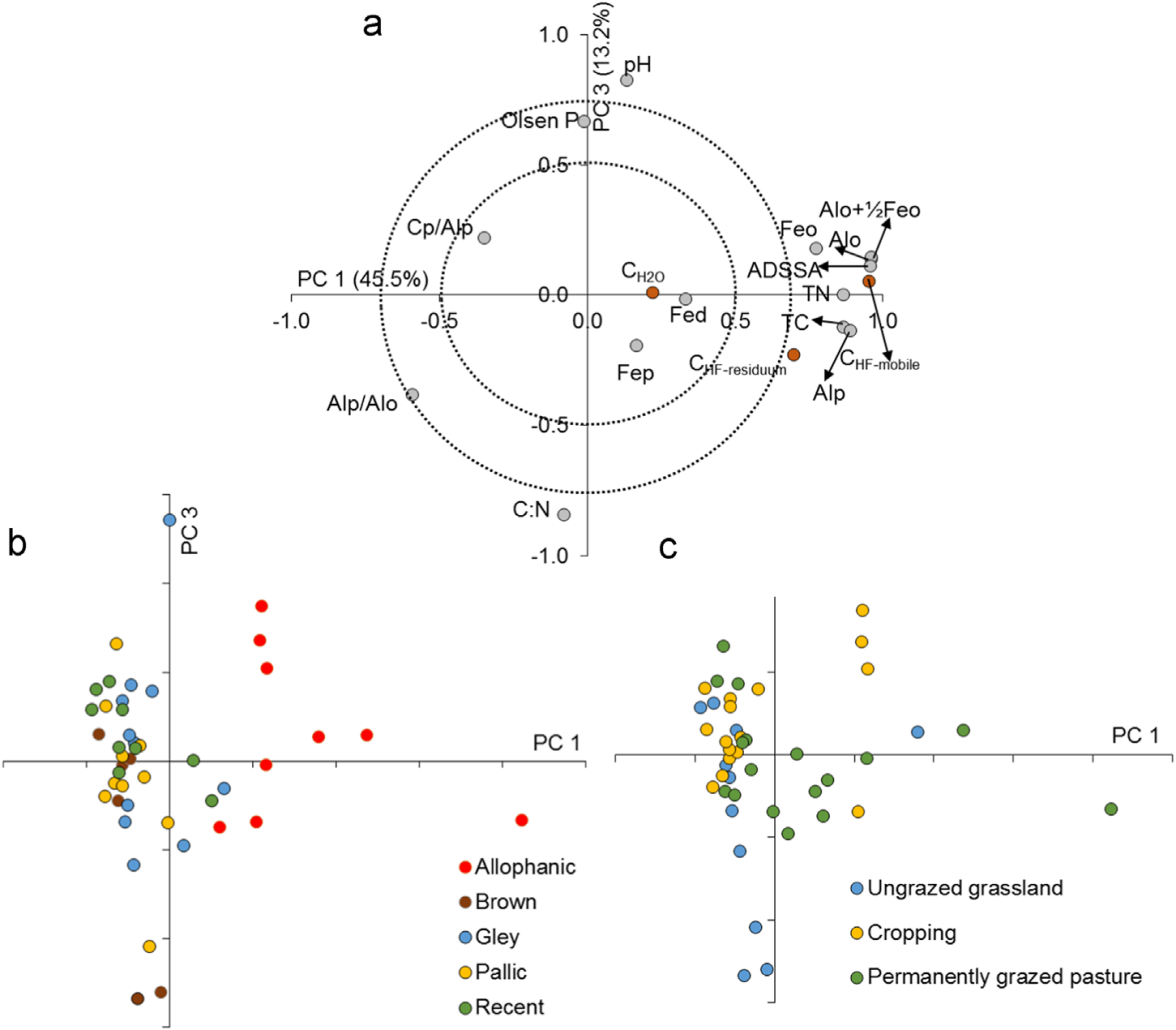
PCA (*PC1 vs. PC3*) of main chemical properties of the set of 45 soils, including C fractions.

**Fig. 8:**
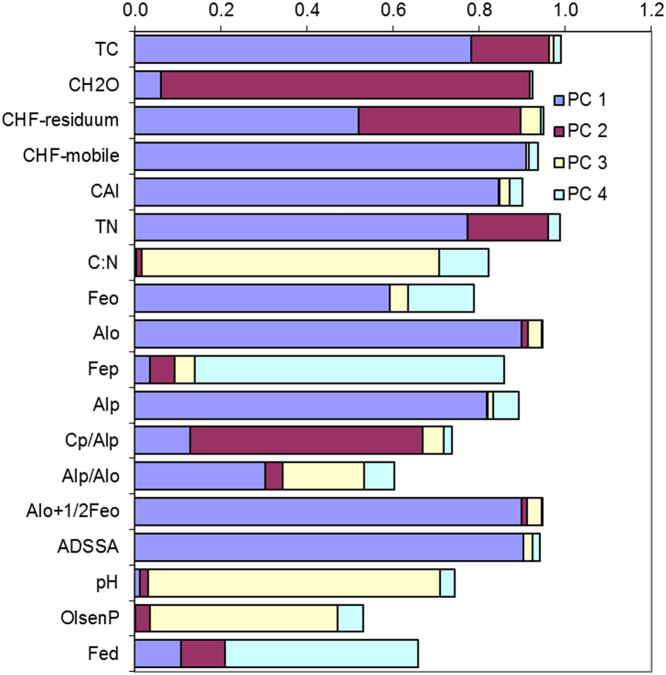
The communalities (square of loadings) of the PCA for the main chemical properties of the set of 45 soils considered, including C fractions.

**Fig. 9:**
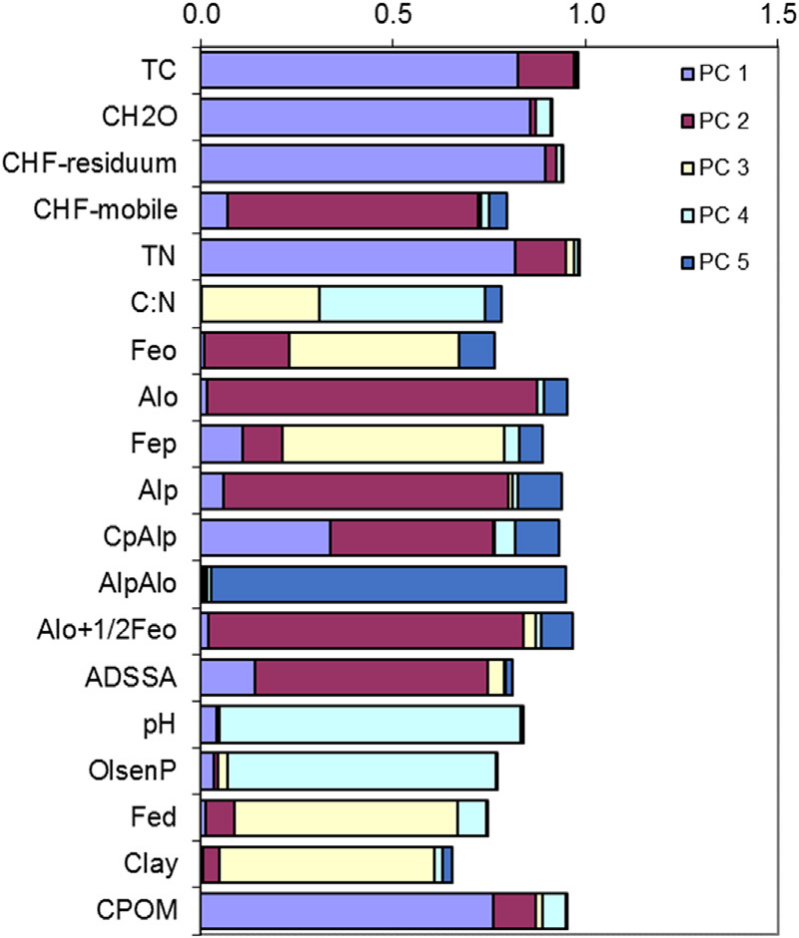
The communalities (square of loadings) of the main chemical properties of non-Allophanic soils.

**Fig. 10:**
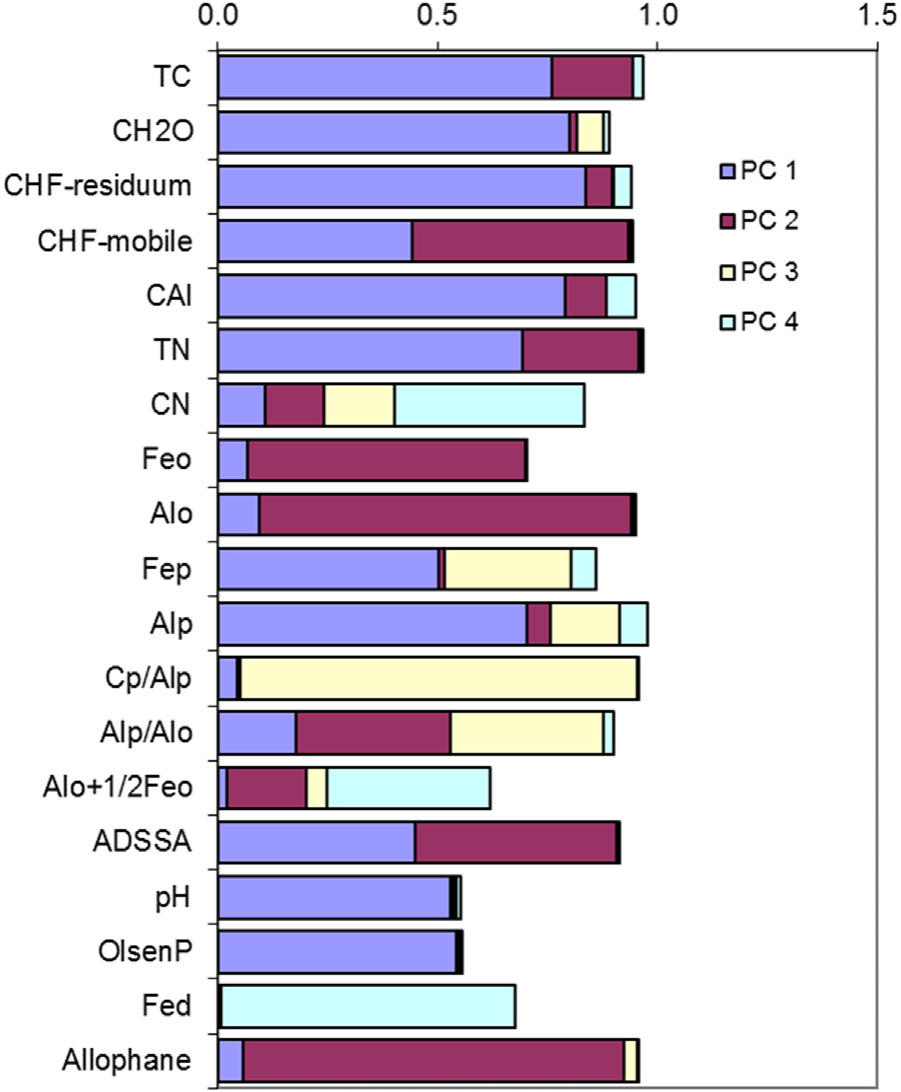
The communalities (square of loadings) of the main chemical properties of Allophanic soils (including the subset of 17 Allophanic soils).

In this report, we present a data set acquired with the use of a chemical fractionation and pyrolysis (Py)-GC/MS on soil organic matter quantity and quality, along with a thorough chemical characterisation of the soils. The soil sampled sites are indicated in Fig. 1. The equivalents of New Zealand soil orders to Soil Taxonomy USDA are provided in Table 1. The main soil properties of a range of soil orders (i.e., Allophanic, Brown, Gley, Pallic and Recent) and land uses (i.e., permanently grazed pasture, ungrazed/unmanaged grasslands, annual cropping) are presented in Table 2. The abundance of inorganic short-range order constituents and organo-Al/Fe complexes (as inferred by Al_o_ + ½Fe_o_ values) and of Al cations associated with organic ligands (as inferred by Al_p_ values) are provided in box plots in Fig. 2a and b. Relative values (%) of different C fractions (C_HF-mobile_, C_Al_, C_HF-residuum_ and C_H2O_) of all soils studied (*n* = 45) are provided in box plots in Fig. 3. The communalities resulting from principal component analysis (PCA) of the main soil properties (excluding the C fractions) are presented in Fig. 4. Box plots of soil pH and Olsen P values of different soil orders and land uses are provided in Fig. 5. Box plots of the ratio of C/N are shown in Fig. 6. The PCA for the main soil properties considered, including the C fractions (C_H2O_, C_HF-residuum_, and C_HF-mobile_), for PC1 and PC3, is shown in Fig. 7. The communalities of these properties are presented in Fig. 8. The communalities of the PCA obtained when the soils were grouped as non-Allophanic and Allophanic (here including the additional set of 17 soils) are represented in Fig. 9, Fig. 10, respectively.

**Table 1:** Correspondence between the New Zealand soil orders studied and the USDA equivalent soil orders [2].

New Zealand soil order	Soil Taxonomy equivalent	
	Predominant order/Diagnostic Characteristics	Major suborders
Allophanic	Andisols	Udands, Aquands
Brown	Inceptisols	Udepts
Gley	Aquic moisture regime	Aquepts, Aquents
Pallic	Alfisols, Inceptisols	Aqualfs, Ustalfs, Udalfs
Recent	Entisols	Psamments, Orthents, Fluvents

**Table 2:** Soil physico-chemical properties.

Code	Region	LU	SO	TC	aC_H2O_	bC_HF-residuum_	cC_HF-mobile_	dC_p_	TN	C:N	eFe_o_	fAl_o_	gFe_p_	hAl_p_	C_p_/Al_p_	Al_p_/Al_o_	Al_o_+½Fe_o_	iADSSA	pH	Olsen P	jFe_d_	Clay	^k^C_POM_
				g/kg											at/at	at/at	g/kg	m^2^/g		mg/kg	g/kg	%	g/kg
1	Waikato	Cropping	Allophanic	39.4	0.9	20.6	17.9	9.8	4.1	9.7	6.7	34.5	1.3	3.1	7.2	0.1	37.8	118	6.8	28.4	12.6	11.6	5.6
2	Waikato	Cropping	Allophanic	52.6	1.2	31.4	20.0	13.6	5.3	10.0	5.9	28.6	1.1	4.4	6.9	0.2	31.5	102	6.1	44.6	10.7	11.8	5.6
3	Waikato	Cropping	Allophanic	40.7	0.9	21.7	18.2	10.1	4.0	10.3	7.8	30.5	1.6	3.3	7.0	0.1	34.4	120	6.4	39.6	11.2	9.6	4.4
4	Waikato	Cropping	Allophanic	55.3	1.2	35.6	18.5	19.4	4.4	12.5	3.3	23.2	1.9	6.1	7.2	0.3	24.8	99	5.8	27.0	29.5	17.3	6.4
5	Canterbury	Cropping	Brown	21.0	0.8	16.4	3.8	6.3	2.0	10.6	2.5	1.9	1.5	1.6	9.1	0.8	3.1	29	6.1	26.2	6.7	23.6	1.8
6	Canterbury	Cropping	Brown	38.9	1.7	23.7	13.6	10.1	3.5	11.1	2.0	2.7	2.5	2.7	8.4	1.0	3.7	39	6.0	36.1	8.3	23.8	4.5
7	Southland	Cropping	Brown	37.8	1.6	26.7	9.6	10.1	3.6	10.6	4.5	3.6	3.3	2.6	8.8	0.7	5.8	44	5.8	25.6	9.6	29.6	3.8
8	Waikato	Cropping	Gley	24.9	1.0	14.1	9.8	5.8	2.5	9.8	5.0	10.9	1.2	2.0	6.6	0.2	13.4	77	7.0	66.6	8.3	21.6	2.0
9	Waikato	Cropping	Gley	28.4	0.8	16.4	11.1	7.1	2.6	10.8	4.6	4.6	2.0	2.1	7.5	0.5	6.9	75	6.2	37.6	8.0	19.2	2.4
10	Canterbury	Cropping	Gley	43.3	1.9	38.1	3.3	4.0	4.1	10.6	5.6	2.2	3.3	1.1	26.4	0.5	5.0	42	5.9	38.4	6.9	34.6	6.0
11	Canterbury	Cropping	Gley	40.2	2.0	32.5	5.7	12.7	3.7	10.8	4.1	3.0	2.5	1.1	22.7	0.4	5.0	41	5.9	22.7	5.7	40.3	4.4
12	Canterbury	Cropping	Pallic	31.2	1.2	23.0	7.0	11.2	2.9	10.6	4.0	3.2	2.3	2.6	7.1	0.8	5.2	34	5.9	30.8	6.0	24.9	3.4
13	Southland	Cropping	Pallic	25.2	1.2	17.9	6.2	6.5	2.2	11.6	3.9	2.1	2.6	1.3	11.2	0.6	4.1	33	6.0	15.2	9.6	23.0	1.9
14	Canterbury	Cropping	Pallic	19.4	0.6	19.0	0.4	5.9	1.7	11.5	3.9	2.1	1.7	1.6	8.2	0.8	4.1	23	5.8	23.6	5.6	19.7	1.9
15	Canterbury	Cropping	Recent	22.2	1.0	17.9	3.4	6.9	2.0	11.2	4.2	3.6	2.0	1.9	8.2	0.5	5.7	28	6.3	36.9	3.4	20.8	3.1
16	Canterbury	Cropping	Recent	26.1	1.1	19.1	6.0	8.9	2.4	10.7	3.7	3.3	2.1	2.1	9.8	0.6	5.1	31	6.0	23.3	8.0	22.7	2.6
17	Hawkes Bay	Cropping	Recent	13.1	0.6	9.7	2.8	1.5	1.4	9.5	3.3	1.9	1.3	0.4	18.8	0.2	3.6	21	5.6	23.6	4.7	15.7	1.4
18	Auckland	Permanently grazed pasture	Allophanic	61.5	1.5	45.0	15.0	12.9	5.2	11.8	5.1	13.3	5.9	7.9	3.7	0.6	15.9	90	6.0	20.0	12.3	24.7	10.8
19	Waikato	Permanently grazed pasture	Allophanic	66.7	2.3	44.1	20.4	18.7	6.4	10.4	5.0	18.5	2.1	6.1	6.9	0.3	21.0	118	5.9	24.0	8.0	12.1	13.2
20	Waikato	Permanently grazed pasture	Allophanic	131.9	2.8	77.8	51.3	36.7	12.3	10.8	17.0	66.8	4.9	11.3	7.3	0.2	75.3	203	5.7	10.3	16.8	12.5	53.4
21	Taranaki	Permanently grazed pasture	Allophanic	92.2	2.0	60.5	31.7	24.6	8.8	10.6	9.9	35.3	2.8	8.8	6.3	0.3	40.3	141	6.2	33.3	12.6	10.0	24.7
22	Auckland	Permanently grazed pasture	Brown	42.0	1.6	29.4	11.0	9.3	3.8	11.0	3.6	3.5	5.1	4.1	5.1	1.2	5.3	59	5.8	24.7	18.7	31.0	8.6
23	Waikato	Permanently grazed pasture	Gley	32.1	1.3	25.1	5.8	7.2	3.2	9.9	7.1	3.0	2.6	1.4	11.7	0.5	6.6	61	6.1	17.4	28.8	25.3	5.1
24	Waikato	Permanently grazed pasture	Gley	65.6	2.4	45.7	17.5	15.9	5.7	11.5	3.6	9.2	1.6	4.1	8.8	0.4	11.0	81	5.9	38.8	2.6	15.4	9.8
25	Waikato	Permanently grazed pasture	Gley	36.8	1.4	28.6	6.8	9.7	3.6	10.1	5.2	4.4	5.4	2.5	8.7	0.6	7.0	71	5.2	6.9	21.4	33.4	8.4
26	Waikato	Permanently grazed pasture	Gley	56.2	1.8	42.2	12.2	16.5	5.2	10.8	7.3	8.0	5.3	3.8	9.7	0.5	11.6	79	5.2	9.7	14.1	24.0	10.8
27	Canterbury	Permanently grazed pasture	Pallic	68.6	4.9	58.0	5.7	17.7	6.7	10.2	4.0	1.1	3.3	0.8	52.9	0.7	3.1	53	5.8	60.1	3.6	23.2	12.8
28	Canterbury	Permanently grazed pasture	Pallic	42.8	1.8	36.9	4.1	11.9	3.8	11.3	4.8	3.5	2.2	2.4	11.1	0.7	5.9	40	6.1	22.3	5.3	23.7	5.4
29	Southland	Permanently grazed pasture	Pallic	58.3	2.9	48.3	7.1	12.4	5.0	11.7	1.1	3.4	1.0	2.4	11.6	0.7	3.9	53	6.0	24.2	1.4	18.9	7.6
30	Southland	Permanently grazed pasture	Pallic	39.1	2.3	26.3	10.5	9.1	3.5	11.1	4.6	3.0	2.7	1.9	10.6	0.6	5.3	45	6.1	26.9	3.0	19.2	4.6
31	Canterbury	Permanently grazed pasture	Recent	37.1	1.8	34.6	0.7	10.2	3.3	11.3	3.9	2.8	1.4	1.5	15.5	0.5	4.8	36	6.2	27.0	6.0	20.7	5.6
32	Southland	Permanently grazed pasture	Recent	65.8	2.4	51.2	12.2	18.1	6.1	10.8	6.6	10.1	3.3	5.0	8.2	0.5	13.4	66	5.8	19.0	11.4	16.4	11.4
33	Southland	Permanently grazed pasture	Recent	30.9	2.1	22.9	6.0	8.0	3.3	9.4	6.0	2.1	2.6	1.4	13.3	0.7	5.1	37	6.0	25.0	9.1	26.1	3.2
34	Southland	Permanently grazed pasture	Recent	57.5	2.3	40.1	15.0	14.6	5.4	10.7	8.9	8.1	4.8	4.6	7.1	0.6	12.5	71	6.0	17.1	11.3	25.5	10.6
35	Taranaki	Ungrazed grassland	Allophanic	80.3	2.7	55.1	22.5	19.9	8.4	9.6	8.2	30.3	1.6	5.2	8.6	0.2	34.4	130	6.1	10.6	11.2	6.4	26.5
36	Canterbury	Ungrazed grassland	Brown	24.8	1.0	20.8	3.1	4.3	1.7	14.5	1.2	2.3	1.3	1.3	7.2	0.6	2.9	30	5.1	7.5	5.8	15.8	3.8
37	Canterbury	Ungrazed grassland	Brown	34.2	1.1	26.1	7.0	8.5	2.4	14.1	5.7	3.6	2.2	2.2	8.7	0.6	6.4	55	4.8	6.8	11.7	19.6	5.6
38	Canterbury	Ungrazed grassland	Gley	32.3	1.4	28.6	2.3	8.1	2.9	11.2	4.0	2.3	1.2	0.8	24.1	0.3	4.3	34	6.1	23.4	6.4	29.4	5.0
39	Canterbury	Ungrazed grassland	Gley	35.8	1.6	28.7	5.6	13.7	3.2	11.1	3.6	2.7	2.3	1.8	17.0	0.7	4.5	41	5.6	9.0	6.3	28.7	5.1
40	Canterbury	Ungrazed grassland	Gley	34.8	1.5	34.8	0.0	10.0	2.9	12.2	1.9	2.7	1.6	1.4	16.4	0.5	3.7	40	5.7	6.0	4.4	20.1	6.5
41	Canterbury	Ungrazed grassland	Pallic	29.5	1.3	25.1	3.1	7.8	2.6	11.5	3.4	2.5	1.8	1.8	9.7	0.7	4.2	31	6.2	20.1	0.5	22.2	4.3
42	Canterbury	Ungrazed grassland	Pallic	29.5	2.2	24.5	2.9	7.1	2.9	10.1	3.0	1.6	1.5	0.6	27.0	0.4	3.1	27	6.1	15.0	7.3	22.7	3.9
43	Canterbury	Ungrazed grassland	Pallic	48.8	1.9	41.6	5.3	9.3	3.6	13.5	2.3	2.4	2.3	1.9	11.2	0.8	3.5	48	5.0	26.6	6.6	17.2	8.9
44	Canterbury	Ungrazed grassland	Recent	28.8	1.4	23.6	3.9	2.6	2.7	10.7	2.0	2.0	1.4	0.7	21.3	0.4	3.0	35	5.8	10.4	5.7	15.6	4.2
45	Southland	Ungrazed grassland	Recent	26.8	2.5	21.9	2.4	4.0	2.8	9.8	5.4	1.7	3.2	1.1	15.6	0.7	4.3	33	5.8	22.6	12.0	24.4	4.8
46	Waikato	Cropping	Allophanic	53.7	1.2	30.9	22.8	13.0	5.3	10.0	8.7	34.0	1.7	5.5	5.4	0.2	3.8	104.1	6.0	39.0	1.1		
47	Waikato	Cropping	Allophanic	50.2	1.1	28.0	22.2	11.6	4.8	10.5	5.9	31.7	1.0	5.0	5.2	0.2	3.5	103.4	6.3	45.9	0.8		
48	Waikato	Pasture	Allophanic	70.2	1.9	44.8	25.3	17.9	7.0	10.1	8.4	32.9	2.2	7.4	5.4	0.2	3.7	117.9	5.9	15.8	1.3		
49	Auckland	Pasture	Allophanic	78.8	1.9	56.1	22.7	24.4	7.1	11.1	6.8	29.7	4.7	9.0	6.1	0.3	3.3	140.8	6.1	20.4	2.0		
50	Auckland	Pasture	Allophanic	67.4	1.5	45.6	21.8	23.4	6.2	10.8	7.5	19.7	6.1	9.4	5.6	0.5	2.3	123.2	5.8	13.0	1.8		
51	Auckland	Pasture	Allophanic	86.2	2.4	60.2	26.0	24.0	7.7	11.2	7.1	25.5	5.5	9.6	5.6	0.4	2.9	149.5	5.9	8.7	0.5		
52	Waikato	Pasture	Allophanic	52.9	1.2	35.1	17.8	16.6	5.2	10.2	7.4	28.0	2.1	5.1	7.3	0.2	3.2	120.1	5.9	7.5	0.9		
53	Waikato	Cropping	Allophanic	59.3	1.7	35.3	24.1	19.6	6.0	9.9	11.4	30.7	3.1	6.2	7.1	0.2	3.6	116.7	5.6	24.3	1.2		
54	Waikato	Cropping	Allophanic	46.7	1.0	26.8	19.9	9.9	4.6	10.3	7.0	33.8	0.8	3.6	6.2	0.1	3.7	127.8	6.5	35.2	1.0		
55	Waikato	Pasture	Allophanic	88.0	3.2	56.4	31.6	26.7	8.2	10.8	4.9	23.7	3.6	7.9	7.6	0.3	2.6	138.6	5.5	26.5	0.2		
56	Waikato	Pasture	Allophanic	73.2	2.6	49.3	23.9	16.9	7.4	9.9	7.0	22.2	2.1	4.5	8.5	0.2	2.6	113.9	6.2	20.8	1.0		
57	Waikato	Pasture	Allophanic	61.6	2.0	38.6	23.0	15.6	5.9	10.5	6.0	21.5	2.6	4.5	7.8	0.2	2.4	102.7	6.1	21.9	0.9		
58	Waikato	Pasture	Allophanic	103.9	2.4	62.0	41.9	27.9	9.5	10.9	9.5	47.6	3.2	9.2	6.8	0.2	5.2	181.4	5.7	6.2	1.3		
59	Waikato	Pasture	Allophanic	55.6	1.5	33.6	22.0	15.2	6.0	9.2	9.1	29.1	2.5	5.1	6.7	0.2	3.4	116.3	5.6	11.9	1.1		
60	Waikato	Pasture	Allophanic	156.1	3.3	103.1	53.0	49.1	13.3	11.8	9.9	51.3	6.8	15.2	7.3	0.3	5.6	194.6	5.5	8.8	1.7		
61	Waikato	Pasture	Allophanic	108.0	2.4	58.4	49.6	28.8	11.0	9.9	8.1	61.6	2.5	9.7	6.7	0.2	6.6	194.6	5.2	9.3	1.3		
62	Taranaki	Pasture	Allophanic	92.2	1.9	60.5	31.7	24.6	8.7	10.5	9.9	35.3	2.8	8.8	6.3	0.2	4.0	140.7	5.6	14.6	1.3		

a C_H2O_: C extracted by water;

b C_HF-residuum_: C in residue recovered after treatment of 2% HF solution;

c C_HF-mobile_: C mobilised in treatment of 2% HF solution;

d C_p_: C extracted by 0.1 M sodium pyrophosphate (pH ~10);

e Fe_o_: ion extracted by 0.1 M acid ammonium oxalate (pH = 3);

f Al_o_: aluminium extracted by 0.1 M acid ammonium oxalate (pH = 3);

g Fe_p_: iron extracted by 0.1 M sodium pyrophosphate (pH ~10);

h Al_p_: aluminium extracted by 0.1 M sodium pyrophosphate (pH ~10);

i ADSSA: specific surface area estimated by air-drying the soils; Fe_d_: ion extracted by dithionite solution;

j C_POM_: C content of the particulate OM fraction (> 53 µm).

**Fig. 11:**
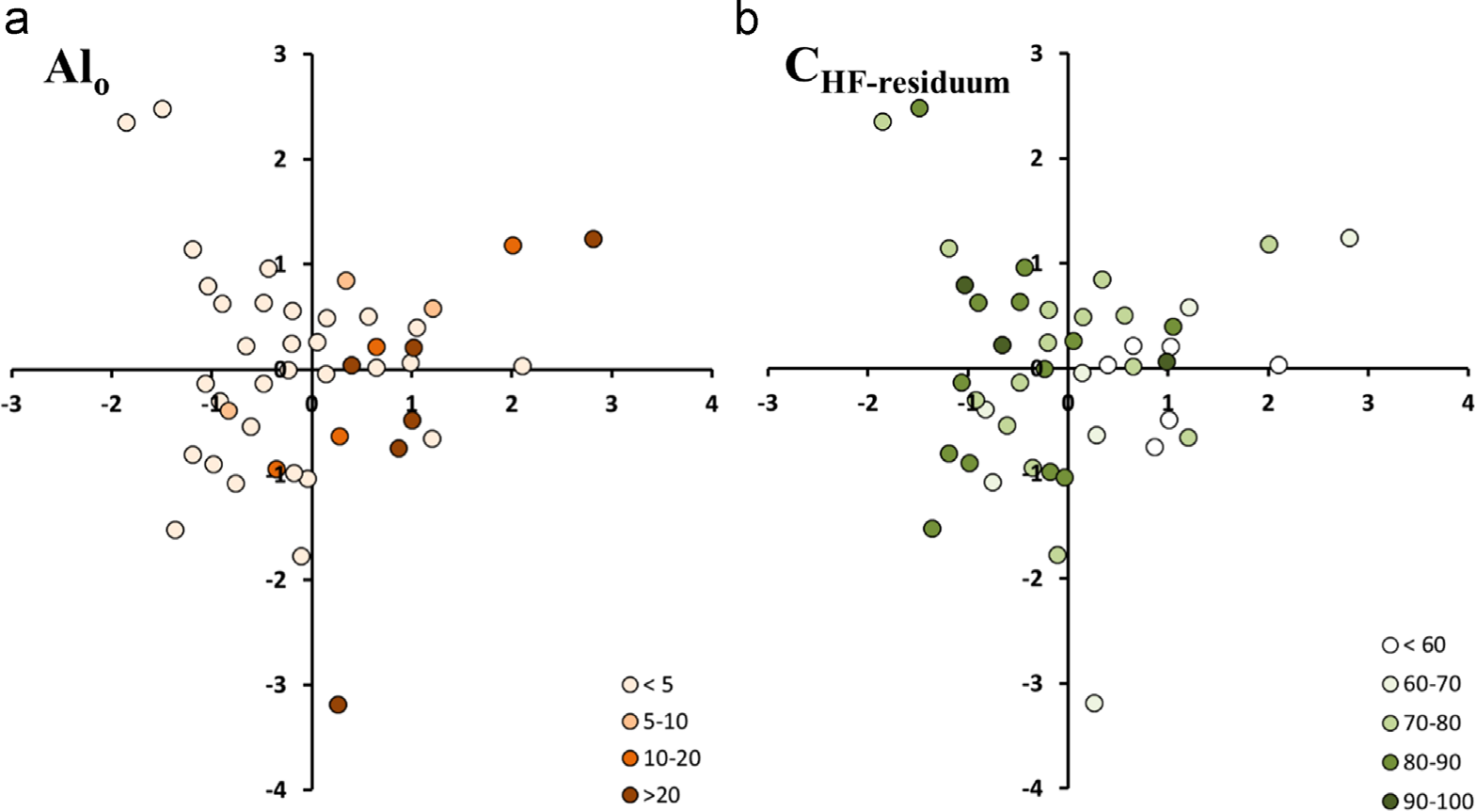
Factor scores of the samples in PC1-PC2 space obtained for the pyrolytic products of all studied soils, classified by (a) Al_o_ content and (b) C_HF-residuum_.

**Fig. 12:**
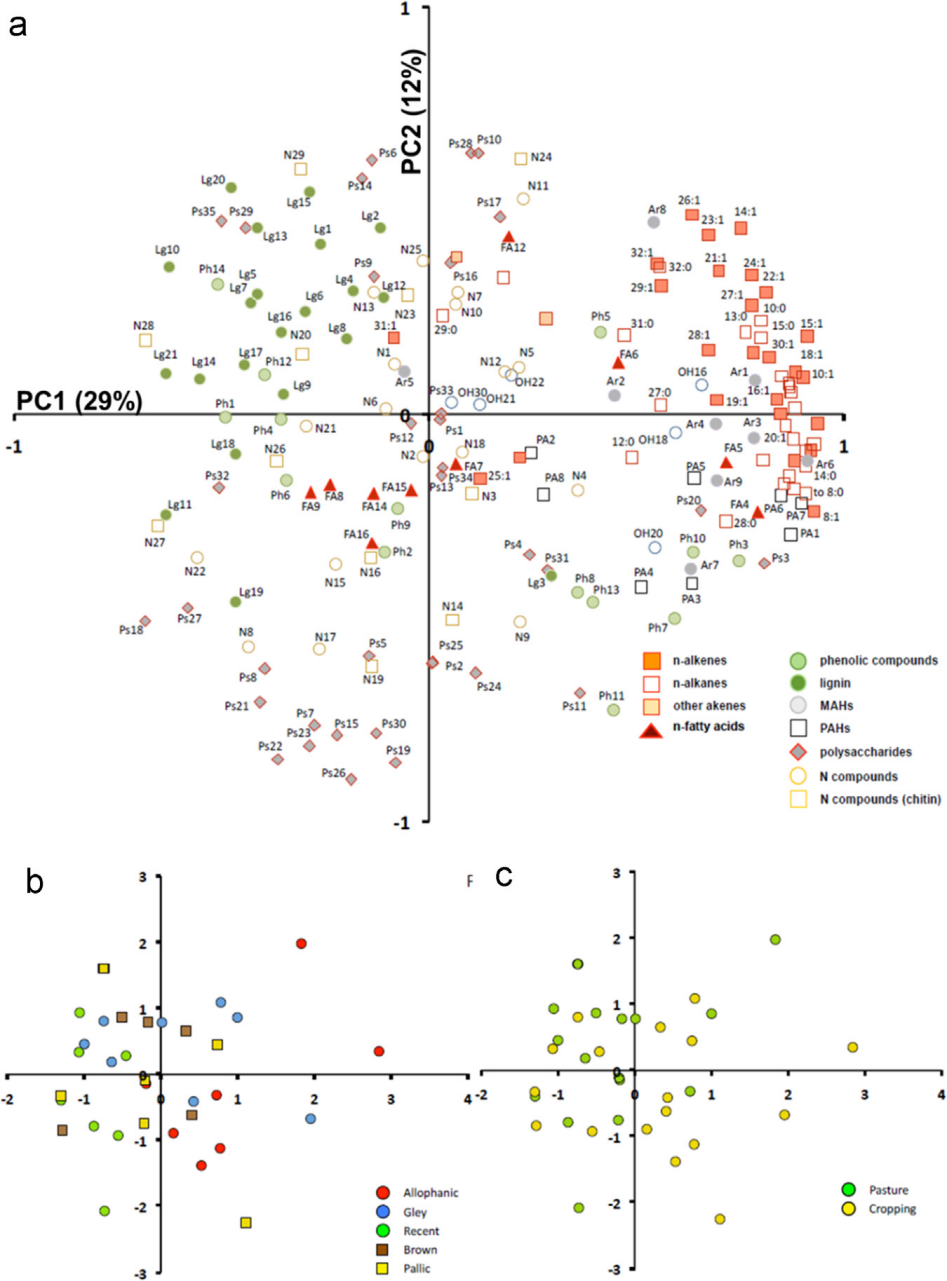
(a) Loadings in PC1-PC2 space of pyrolytic products for the soils under *cropping and permanently grazed pasture*; (b) factor scores of the samples in PC1-PC2 space obtained for the soils under cropping and permanently grazed pasture, classified by soil order (i.e. Allophanic, Pallic, Gley, Brown and Recent soils); (c) scores of the samples in the PC1-PC2 space obtained for the soils under cropping vs. permanently grazed pasture.

The list of pyrolysis products, molecular mass (M+), fragment ion used (*m*/*z*) and average retention time (RT) are presented in Table 3; and the average relative abundances (% TQPA) of pyrolytic compounds, grouped according to their possible origins, obtained for different soil orders (i.e. Allophanic, Pallic, Gley, Brown and Recent soils) under different land use (permanently grazed pasture, ungrazed/unmanaged grasslands, annual cropping) are shown in Table 4. The scores of the PCA carried out for the OM fingerprints obtained through Py-GC/MS for the 45 soils were classified as either Al_o_ (Fig. 11a) or C_HF-residuum_ (Fig. 11b). The loadings of F1-F2 from PCA of pyrolytic products of soils under permanently grazed pasture and cropping vegetation are shown in Fig. 12a, and the scores grouped by soil orders and land uses are shown in Fig. 12b and c, respectively.

**Table 3:** Pyrolysis product list, molecular mass (M+), fragment ion used (m/z) and average retention time (RT).

Code	Group	Compound	M+	*m*/*z*	RT
8:1 to 32:1	n-Alkenes	C8 alkene to C32 alkene	–	55 + 69	–
8:0 to 33:0	n-Alkanes	C8 alkane to C33 alkane	–	57 + 71	–
ene1	Other alkenes	Alkene	*	55 + 69	28.373
ene2	Other alkenes	Alkene	*	55 + 69	28.611
OH16	*n*-Alkanols	C16 Alkanol	242	55 + 69	31.49
OH18	*n*-Alkanols	C18 Alkanol	270	55 + 69	35.398
OH20	*n*-Alkanols	C20 Alkanol	298	55 + 69	38.998
OH21	*n*-Alkanols	C21 Alkanol	312	55 + 69	40.698
OH22	*n*-Alkanols	C22 Alkanol	326	55 + 69	42.307
OH30	*n*-Alkanols	C30 Alkanol	438	55 + 69	53.498
FA4	*n*-Fatty acids	C4 Fatty acid	88	60 + 73	4.265
FA5	*n*-Fatty acids	C5 Fatty acid	102	60 + 73	6.44
FA6	*n*-Fatty acids	C6 Fatty acid	116	60 + 73	9.082
FA7	*n*-Fatty acids	C7 Fatty acid	130	60 + 73	11.865
FA8	*n*-Fatty acids	C8 Fatty acid	144	60 + 73	14.64
FA9	*n*-Fatty acids	C9 Fatty acid	158	60 + 73	17.257
FA12	*n*-Fatty acids	C12 Fatty acid	200	60 + 73	24.09
FA14	*n*-Fatty acids	C14 Fatty acid	228	60 + 73	29.073
FA15	*n*-Fatty acids	C15 Fatty acid	242	60 + 73	31.14
FA16	*n*-Fatty acids	C16 Fatty acid	256	60 + 73	33.148
Ph1	Phenols	Phenol	–	66 + 94	–
Ph2	Phenols	Phenol, 3-methyl-	108	107 + 108	10.948
Ph3	Phenols	Acetophenone	120	105 + 77	11.198
Ph4	Phenols	Phenol, 3/4-methyl	108	107 + 108	11.565
Ph5	Phenols	Phenol, 2,3-dimethyl-	122	107 + 122	13.407
Ph6	Phenols	Phenol, 2,6-dimethyl-	122	107 + 122	13.665
Ph7	Phenols	Acetophenone, 4-hydroxy	136	121 + 136	14.015
Ph8	Phenols	Phenol, 2-ethyl	122	107 + 122	14.215
Ph9	Phenols	Phenol, 3-ethyl	122	107 + 122	14.282
Ph10	Phenols	Phenol, 4-ethyl	122	107 + 122	14.55
Ph11	Phenols	Phenol, 5-ethyl	122	107 + 122	14.996
Ph12	Phenols	Catechol	110	64 + 110	15.418
Ph13	Phenols	5-Ethyl, 2-methyl phenol	136	121 + 136	16.244
Ph14	Phenols	1,2-Benzenediol, 3-methoxy (3-methoxy catechol)	140	125 + 140	16.907
Lg1	Lignin	Guaiacol (phenol, 2-methoxy-)	124	109 + 124	11.89
Lg2	Lignin	4-Methylguaiacol	138	123 + 138	14.915
Lg3	Lignin	4-Vinylphenol	120	91 + 120	15.74
Lg4	Lignin	4-Ethylguaiacol	152	137 + 152	17.357
Lg5	Lignin	4-Vinylguaiacol	150	135 + 150	18.323
Lg6	Lignin	Phenol, 4-(2-propenyl)	134	133 + 134	19.082
Lg7	Lignin	Syringol (phenol, 2,6-dimethoxy-)	154	139 + 154	19.29
Lg8	Lignin	4-(Prop-1-enyl) guaiacol	164	77 + 164	19.477
Lg9	Lignin	4-(Prop-2-enyl) guaiacol, *trans*	164	149 + 164	20.813
Lg10	Lignin	4-Methylsyringol	168	153 + 168	21.79
Lg11	Lignin	4-(Prop-2-enyl) guaiacol, *cis*	164	149 + 164	21.865
Lg12	Lignin	4-Acetylguaiacol	166	151 + 166	22.79
Lg13	Lignin	Propan-2-one guaiacol	180	137 + 180	23.897
Lg14	Lignin	4-Vinylsyringol	180	165 + 180	24.69
Lg15	Lignin	4-Propanedione guaiacol	180	151 + 180	25.129
Lg16	Lignin	4-(Prop-2-enyl) syringol, *trans*	194	91 + 194	25.586
Lg17	Lignin	4-(Prop-2-enyl) syringol, *cis*	194	91 + 194	27.79
Lg18	Lignin	4-Acetylsyringol	196	181 + 196	28.523
Lg19	Lignin	4-(3-Hydroxy-1-propenyl) guaiacol (coniferyl alcohol)	180	137 + 180	28.64
Lg20	Lignin	4-(Propan-2-one) syringol	210	167 + 210	29.343
Lg21	Lignin	Alpha-beta-diguaiacylacylethene	272	272 + 273	44.842
Ar1	MAHs	Benzene	78	77 + 78	2.407
Ar2	MAHs	Toluene	92	91 + 92	3.698
Ar3	MAHs	Ethylbenzene	106	91 + 106	5.582
Ar4	MAHs	1,2-Dimethylbenzene	106	91 + 106	5.751
Ar5	MAHs	Styrene	104	78 + 104	6.299
Ar6	MAHs	1,3-Dimethylbenzene	106	91 + 106	6.348
Ar7	MAHs	Benzaldehyde	106	77 + 106	8.132
Ar8	MAHs	Trimethyl benzene	120	105 + 120	9.865
Ar9	MAHs	Benzene, 1-propenyl	118	117 + 118	10.033
PA1	PAHs	Indene	116	115 + 116	10.548
PA2	PAHs	Naphthalene, 1,2-dihydro	130	129 + 130	13.639
PA3	PAHs	1H-Indene, 3-methyl	130	115 + 130	13.639
PA4	PAHs	1H-Indene, 2-methyl	130	115 + 130	13.811
PA5	PAHs	Naphthalene	128	128	14.632
PA6	PAHs	Naphthalene, 1-methyl	142	141 + 142	17.748
PA7	PAHs	Naphthalene, 2-methyl	142	141 + 142	18.221
PA8	PAHs	Phenanthrene/anthracene, x-ethyl	206	191 + 206	23.423
Ps1	Polysaccharides	Furan, 2-methyl-	82	53 + 82	1.99
Ps2	Polysaccharides	Acetic acid	60	45 + 60	2.057
Ps3	Polysaccharides	1,3-Cyclopentadiene, x-methyl	80	77 + 80	2.494
Ps4	Polysaccharides	Furan, 2,5-dimethyl	96	95 + 96	2.848
Ps5	Polysaccharides	Furan, 3-methyl	82	53 + 82	3.34
Ps6	Polysaccharides	(2*H*)-Furan-2-one	84	54 + 84	4.19
Ps7	Polysaccharides	2-Furaldehyde	96	95 + 96	4.507
Ps8	Polysaccharides	2,5-Furandione	98	54 + 98	4.851
Ps9	Polysaccharides	3-Furaldehyde	96	95 + 96	4.907
Ps10	Polysaccharides	2-Cyclopentene-1,4-dione	96	68 + 96	6.098
Ps11	Polysaccharides	2-Cyclopenten-1-one, 2-methyl	96	67 + 96	6.673
Ps12	Polysaccharides	2-Acetylfuran	110	95 + 110	6.803
Ps13	Polysaccharides	2-Furaldehyde, 5-methyl	110	109 + 110	6.897
Ps14	Polysaccharides	Dihydro-3-methylene-2(3*H*)-furanone	98	68 + 98	7.107
Ps15	Polysaccharides	2,3-Dihydro-5-methylfuran-2-one	98	55 + 98	7.165
Ps16	Polysaccharides	2(5*H*)-Furanone, 5-methyl	98	55 + 98	7.537
Ps17	Polysaccharides	2-Furaldehyde, 5-methyl	110	109 + 110	8.215
Ps18	Polysaccharides	4-Hydroxy-2,6-dihydro-2H-pyran-2-one	114	114 + 58	9.207
Ps19	Polysaccharides	3-Hydroxy-2-methyl-2-cyclopenten-1-one	112	55 + 112	10.073
Ps20	Polysaccharides	2,3-Dimethylcyclopent-2-en-1-one	110	67 + 110	10.399
Ps21	Polysaccharides	Dianhydrorhamnose	128	113 + 128	10.448
Ps22	Polysaccharides	2,5-Dimethyl-4-hydroxy-3(2*H*)-furanone	128	57 + 128	11.766
Ps23	Polysaccharides	2H-Pyran-4-one, 3-hydroxy-2-methyl-	126	71 + 126	12.612
Ps24	Polysaccharides	Sugar (unknown)	*	71 + 87	13.299
Ps25	Polysaccharides	Sugar (unknown)	*	71 + 87	13.907
Ps26	Polysaccharides	1-Deoxy-2,4-methylene-3,5-anhydro-D-xylitol?	130	69 + 100	14.519
Ps27	Polysaccharides	3,5-Dihydroxy-2-methyl-4H-pyran-4-one	142	68 + 142	14.799
Ps28	Polysaccharides	1,4:3,6-Dianhydro-alpha-D-glucopyranose	144	57 + 69	15.365
Ps29	Polysaccharides	1,4,-Dideoxy-D-Glycero-Hex-1-enopyranos-3-ulose	144	87 + 144	18.065
Ps30	Polysaccharides	2(5*H*)-Furanone, -dimethyl-	112	69 + 97	18.449
Ps31	Polysaccharides	L-Glucose, 6-deoxy-3-O-methyl-?	178	74 + 59 + 103	19.715
Ps32	Polysaccharides	Levogalactosan	162	60 + 73	19.865
Ps33	Polysaccharides	Arabinopyranoside?	*	74 + 101	21.24
Ps34	Polysaccharides	Levomannosan	162	60 + 73	21.848
Ps35	Polysaccharides	Levoglucosan	162	60 + 73	23.332
N1	N-compounds	Pyridine	79	52 + 79	3.357
N2	N-compounds	Pyrrole	67	67	3.515
N3	N-compounds	Acetamide	59	59	3.94
N4	N-compounds	Pyridine, 2-methyl	93	66 + 93	4.598
N5	N-compounds	1*H*-Pyrrole, 2-methyl	81	80 + 81	5.099
N6	N-compounds	1*H*-Pyrrole, 3-methyl	81	80 + 81	5.31
N7	N-compounds	Pyridine, 3-methyl	93	93 + 66	5.583
N8	N-compounds	1*H*-Imidazole, 4,5-dihydro-2-methyl (Lysidine)	84	55 + 84	6.864
N9	N-compounds	1*H*-pyrrole, 3-ethyl	95	80 + 95	7.223
N10	N-compounds	Pyridine, 2,5-dimethyl	107	107 + 106	7.348
N11	N-compounds	1*H*-Pyrrole, 2,4-dimethyl	95	94 + 95	7.532
N12	N-compounds	1*H*-Pyrrole, 1-ethyl	95	80 + 95	7.773
N13	N-compounds	1*H*-Pyrrole-2,5-dione	97	54 + 97	9.096
N14	N-compounds	1*H*-Pyrrole-2-carboxaldehyde	95	66 + 95	9.646
N15	N-compounds	Pyrrole, 4-ethyl-2-methyl	109	94 + 109	11.098
N16	N-compounds	2-Hydroxypyridine	95	67 + 95	12.855
N17	N-compounds	1*H*-pyrazole, 3-methyl	82	81 + 82	13.051
N18	N-compounds	Benzonitrile, 2-methyl	117	116 + 117	13.332
N19	N-compounds	3-Acetamidofuran	125	83 + 125	16.132
N20	N-compounds	Picolinamide	122	79 + 122	16.801
N21	N-compounds	Indole	117	90 + 117	17.765
N22	N-compounds	1*H*-Indole, 3-methyl	131	130 + 131	20.232
N23	N-compounds	Acetamide, N-(2-hydroxyphenyl)	109	80 + 109	20.421
N24	N-compounds	Acetamide, N-(2,4-dihydroxyphenyl)	167	125 + 167	23.34
N25	N-compounds	Diketodipyrrole	186	93 + 186	27.923
N26	N-compounds	Diketopiperazine derivative, Pyrrolo [1,2-a]pyrazine-1,4-dione, hexahydro-3-(2-methylpropyl)	210	70 + 154	30.273
N27	N-compounds	Diketopiperazine derivative	210	70 + 154	32.226
N28	N-compounds	Diketopiperazine derivative	210	70 + 154	32.537
N29	N-compounds	Diketopiperazine derivative	250	70 + 194	32.74

(* indicates unknown molecular mass; MAHs, monocyclic aromatic hydrocarbons; PAHs, polycyclic aromatic hydrocarbons)

**Table 4:** Average relative abundances (% TQPA) of pyrolytic compounds, grouped according to possible origins, obtained for different soils orders (i.e. Allophanic, Pallic, Gley, Brown and Recent soils) under different land use (ungrazed grassland, cropping, and pasture).

	Allophanic soil			Gley Soil			Recent soil			Brown soil			Pallic soil		
	Ungrazed	Crop.	Grazed	Ungrazed	Crop.	Grazed	Ungrazed	Crop.	Grazed	Ungrazed	Crop.	Grazed	Ungrazed	Crop.	Grazed
Polysaccharides (%TQPA)	45.6	52.6	51.2	60.5	53.6	55.6	52.8	54.9	56.5	64.3	54.4	48.5	54.8	53.9	54.4
Anhydrosugars (%Ps)	38.4	48.3	50.3	56.4	51.6	51.1	48.4	50.0	49.4	59.3	49.3	51.7	51.2	50.7	51.4
4-Hydroxy-5,6-dihydro-(2H)-pyran-2-one (%Ps)	4.5	2.8	3.0	3.6	2.9	2.8	6.0	4.9	5.2	3.3	3.8	3.8	4.9	4.4	4.2
Sum Nitrogen (%TQPA)	15.9	12.9	12.3	11.1	12.1	11.9	12	12.4	11.9	6.2	12.4	12.2	12.0	11.7	10.3
Intact protein (%N)	9.6	6.0	8.5	7.9	7.8	8.4	9.5	8.7	8.4	7.6	8.4	8.9	9.6	9.1	9.1
Chitin markers (%TQPA)	5.0	5.5	4.5	4.3	4.4	4.7	4.8	4.3	4.4	2.3	4.5	4.4	3.9	3.8	3.6
Sum MCCs (%TQPA)	12.5	14.9	15.6	9.1	13.8	12.3	9.3	10.8	11.2	7.8	12.5	13.1	9.5	12.8	13.3
n-Alkenes (%TQPA)	5.1	5.4	5.6	3.0	4.7	4.1	2.5	3.2	3.7	2.6	3.8	5.5	3.3	5.0	4.2
n-Alkanes (%TQPA)	5.3	7.6	8.6	3.9	7.3	6.5	4.7	5.8	5.9	3.1	6.8	6.4	4.5	6.1	7.1
n-Fatty Acids (%TQPA)	1.8	1.2	1.0	1.8	1.2	1.2	1.7	1.3	1.3	1.7	1.4	0.8	1.4	1.1	1.6
n-Alkanols (%TQPA)	0.1	0.5	0.3	0.3	0.5	0.4	0.2	0.3	0.2	0.3	0.4	0.4	0.2	0.5	0.3
Sum Lignin (%TQPA)	7.5	5.7	6.3	7.0	6.3	6.4	9.4	7.3	7.0	6.7	6.5	6.8	8.8	7.1	8.1
Guaiacols (G, %Lg)	60.3	52.8	58.2	63.3	54.4	59.5	61.1	56	60.7	61	60.4	59.8	63.5	55	58.6
Syringols (S, %Lg)	14.3	12.7	14.7	13	14.3	15.2	17.3	16.0	16.0	12.2	15.2	17.3	12.4	14.3	14.1
Hydroxyphenyl (H, %Lg)	25.4	34.5	27.1	23.7	31.2	25.4	21.5	27.9	23.3	26.9	24.3	22.9	24	30.7	27.3
S/G	0.2	0.2	0.3	0.2	0.3	0.3	0.3	0.3	0.3	0.2	0.3	0.3	0.2	0.3	0.2
C3G/GT	0.12	0.1	0.1	0.09	0.08	0.09	0.13	0.1	0.11	0.11	0.11	0.08	0.11	0.1	0.12
C3S/ST	0.2	0.22	0.21	0.19	0.18	0.19	0.2	0.21	0.21	0.16	0.17	0.19	0.16	0.17	0.33
H/(S + G)	0.34	0.53	0.38	0.31	0.46	0.34	0.27	0.39	0.31	0.37	0.32	0.3	0.32	0.48	0.38
Phenols (%TQPA)	10.2	5.9	7.2	6.4	6.5	6.9	8.0	7.2	7.4	4.6	6.5	6.9	7.9	7.0	7.5
MAHs (%TQPA)	7.2	7.0	6.6	5.3	6.9	6.1	5.6	6.7	5.4	3.9	6.8	6.1	5.8	6.4	5.5
PAHs (%TQPA)	1.0	1.0	0.9	0.7	0.9	0.8	0.7	0.8	0.8	0.6	0.9	0.8	0.8	1.0	0.8

## 2 Experimental design, materials and methods

### 2.1 Soil samples

A representative subset of 45 soils was selected from a larger project in which soils (0–15 cm depth) were collected from New Zealand׳s major agricultural regions (Waikato, Taranaki, Hawkes Bay, Canterbury, Auckland, Southland; Fig. 1) [3]. These included 9 Allophanic soils, 6 Brown soils, 11 Gley soils, 10 Pallic soils and 9 Recent soils under different land uses: permanently grazed pasture (i.e. dairy and drystock; 17 soils), continuous cropping (i.e. arable; 17 soils) or unmanaged and ungrazed grassland (11 soils). An additional subset of 17 Allophanic soils was included when doing PCA of only Allophanic soils to make a total of 26 Allophanic soils; of these 26, 17 were permanently grazed pasture, and 8 under continuous cropping. Only one Allophanic soil under ungrazed grasslands was included in the study. Details on soil sampling are provided in McNally et al. [3].

### 2.2 Soil characterisation

Physico-chemical characteristics (including pH, clay content, short–range ordered materials, organo-Al complexes, total N and C, etc) of the soils were determined following the method described by Shen et al. [1]. The specific surface area (ADSSA) and particle size distribution was determined following soil dispersion by sonication (60 s at 64 J s^−1^) [3]. Carbon was fractionated into cold + hot water-soluble C (C_H2O_), sodium pyrophosphate extractable C (C_p_), C recovered in the residuum after HF treatment (C_HF-residuum_), and C not recovered as calculated by difference from total C (C_HF-mobile_). Further details of the methodology are described in Shen et al. [1]. Soil OM fingerprints were determined by using pyrolysis-GC/MS after treatment with 2% HF solution. Details of the HF treatment, the machine setup and the quantification of the pyrolysis products using GCMSsolution version 2.17 software (Shimadzu) can be found in Shen et al. [1].

### 2.3 Data analysis

The statistical software R version 3.2.2 was used to make box plots of the various C fractions and chemical properties of the soils studied. One-way ANOVA with a Tukey HSD test was conducted to evaluate statistical differences (‘***’ 0.001, ‘**’ 0.01, ‘*’ 0.05, ‘.’ 0.1). Principal component analysis of the C fractions and their chemical properties, and of the pyrolysis products of OM compounds of the studied soils was performed using SPSS software for Windows (IBM SPSS Statistics version 24.0; IBMCorp, 2016). More details are provided in Shen et al. [1].
